# SIX5-activated LINC01468 promotes lung adenocarcinoma progression by recruiting SERBP1 to regulate SERPINE1 mRNA stability and recruiting USP5 to facilitate PAI1 protein deubiquitylation

**DOI:** 10.1038/s41419-022-04717-9

**Published:** 2022-04-06

**Authors:** Yuan Yuan, Danyang Zhou, Feifei Chen, Zhenhua Yang, Wei Gu, Kai Zhang

**Affiliations:** Department of Respiratory Medicine, Nanjing First Hospital, Nanjing Medical University, 210006 Nanjing, Jiangsu China

**Keywords:** Cancer, Biomarkers

## Abstract

Increasing research has uncovered the involvement of long noncoding RNAs (lncRNAs) in the progression of multiple cancers including lung adenocarcinoma (LUAD). RT-qPCR and western blot were done to measure RNAs and proteins. Functional assays assessed LUAD cell biological behaviors under knockdown or overexpression of LINC01468, SIX5, SERBP1 or SERPINE1, and the specific function of those genes in regulating LUAD progression was evaluated via animal experiments. Supported by bioinformatics analysis, the interaction among genes was verified via mechanism assays. Upregulation of LINC01468 in LUAD tissues and cells as well as its association with poor clinical outcome was predicted. LINC01468, transcriptionally activated by SIX5, could strengthen proliferative, migratory and invasive abilities of LUAD cells. The oncogenic role of LINC01468 was further validated via animal experiments. SIX5 was a positive transcription regulator of LINC01468 and could exacerbate LUAD cell malignant behaviors. LINC01468 could recruit SERBP1 to enhance SERPINE1 mRNA stability and interact with USP5 to affect PAI1 protein ubiquitination. The oncogenic role of SERBP1 and SERPINE1 was also confirmed. Rescue experiments finally verified LINC01468 modulated proliferation, migration and invasion of LUAD cells via upregulation of SERPINE1. Our observations could contribute to deeper understanding of LUAD.

## Background

Lung cancer is one of the leading causes of cancer-associated death worldwide [1]. Approximately, patients at an advanced stage account for 50% of lung cancer cases [2], most of which finally suffer from poor prognosis due to the occurrence of liver or brain metastases [3]. Smoking and the air pollution are the main causes of lung cancer [4]. In the last decades, despite great progress in early diagnosis and therapeutic methods [5], 5-year survival rate of lung cancer patients is still relatively poor [4, 5]. Lung adenocarcinoma (LUAD) is the commonest histological type of lung cancer. Hence, it is imminent to find out molecular mechanisms implicated in LUAD tumorigenesis and progression so as to discover new treatment strategies.

Recently, long noncoding RNAs (lncRNAs) with more than 200 nt have gotten extensive attention due to their post-transcriptional regulation from nuclear organization to epigenetic modification of regulation [6, 7]. According to the distance from their location to the coding genes, lncRNAs are divided into five types such as sense, antisense, intergenic, bidirectional and intronic [8]. Accumulating reports have uncovered the crucial involvement of long intergenic noncoding RNAs (lincRNAs), members of lncRNAs whose transcript units locate between the protein-coding genes, in modulating various cellular biological processes in a wide range of diseases [9], including human cancers [10–18]. LincRNAs have also been suggested as crucial effectors in regulating LUAD cell biological behaviors and LUAD development. For instance, LINC01614, upregulated in LUAD tissues and cell lines, has been validated to strengthen the proliferative ability of LUAD cells via miR-217/FOXP1 [19]. LINC00680 has been proved to facilitate LUAD cell cycle via GATA6/SOX12 [20]. In the present study, supported by bioinformatics analysis, a novel lincRNA named lncRNA activating regulator of DKK1 (LINC01468) was identified to be upregulated in lung cancer and have close association with patients’ prognosis. There is limited literature revealing the participation of LINC01468 in malignancy progression, and therefore, the specific function of LINC01468 in LUAD and the underlying regulatory mechanism deserve to be elucidated.

Transcription factors have been reported as crucial regulators of the transcriptional activity of their downstream target genes [21]. More specifically, STAT3 has been validated to transcriptionally activate lnc‑IGF2‑AS and lnc‑7SK [22]. HNF4α has been suggested as a transcriptional suppressor of lnc-APUE [23]. In this study, after confirming LINC01468 was upregulated in LUAD tissues and cell lines, we also aimed to explore the putative transcription factor essential for LINC01468 transcriptional activity.

Protein–RNA interactions are keys to various cellular processes, and their deregulation is implicated in pathologies, and lncRNAs have been discovered to bind to one or more proteins to exert their functions [24]. Based on previous study, the cellular distribution of lncRNAs is essential to their molecular mechanisms [25]. Nuclear RNAs most often function as key regulators for transcriptional programs and subcellular structures while cytoplasmic lncRNAs are suggested to influence mRNA turnover, translation and protein stability via interaction with RNA-binding proteins (RBPs) [26]. More specifically, B4GALT1-AS1 has been ascertained to enhance YAP mRNA stability via recruiting HuR in osteosarcoma cells [27]. ZFPM2-AS1 has been proved to destabilize ZFPM2 mRNA via interaction with UPF1 in LUAD cells [28]. In this study, after determining the main distribution of LINC01468 in cell cytoplasm, we also hypothesized the involvement of certain RBPs in LINC01468 modulation mechanism in LUAD cells. Supported by bioinformatics analysis and mechanism assays, LINC01468 was discovered to possess strong affinity with two RBPs, namely SERPINE1 mRNA binding protein 1 (SERBP1) and ubiquitin specific peptidase 5 (USP5). Based on description on GeneCards, SERBP1 might function in the regulation of mRNA stability and post-translational modifications for USP5 are also mentioned. On this basis, further experiments were done to fathom out the influences of LINC01468-RBP interaction on mRNA and protein stability of the downstream gene.

In general, the main aim in current study was to unveil the specific function of LINC01468 in the progression of LUAD and elucidate the underlying modulation mechanism with transcription factor and RBPs (SERBP1 and USP5) taken into consideration. The findings might offer a novel insight into the inner molecular mechanism implicated in LUAD progression.

## Materials and methods

### Tissue samples

LUAD tissues and adjacent normal tissues were collected from 60 LUAD patients who were diagnosed at Nanjing First Hospital, Nanjing Medical University. Patients enrolled in this study were not treated with any type of therapies before surgical resection. Patients had signed written informed consent. This study has been approved by the Ethics Committee of Nanjing First Hospital, Nanjing Medical University.

### Cell culture

LUAD cell lines including A549, Calu3 (Korean Cell Line Bank, Seoul, Korea), SPC-A1 (Chinese Academy of Sciences, Shanghai, China), and H1299 (University of Pennsylvania, Philadelphia, PA, USA) were cultured in RPMI-1640 medium (WelGENE, Seoul, Korea) supplemented with 10% fetal bovine serum (FBS; Gemini Bio-Products, USA). BEAS-2B cells were purchased from ATCC and cultured in DMEM medium containing 10% FBS and 1% Penicillin-Streptomycin. All cells were maintained in the incubator at 37 °C with 5% CO_2_.

### Gene knockdown and overexpression

Small interfering RNAs (siRNAs) were obtained to generate siRNAs targeting LINC01468, SIX5, SERBP1, USP5, SERPINE1 and other 12 putative transcription factors of LINC01468. Short hairpin RNAs (shRNAs) were used to construct sh-LINC01468 and sh-SERPINE1 specially used in animal experiments. Additionally, the full-length of LINC01468, SIX5, SERBP1, USP5, SERPINE1 and other 13 putative transcription factors of LINC01468 was, respectively, cloned into pcDNA3.1 for overexpression of indicated genes. siCtrl, shCtrl and Ctrl were taken as negative controls. Lipofectamine 2000 was applied for plasmid transfection. Cells were collected for assays 48 h post transfection.

### Cell counting kit-8 (CCK-8) assay

For CCK-8 assays, cells at a density of 1000 cells/well were cultured in a 96-well plate. Plates were then incubated for 2 h after each well was added with 10 μL CCK-8 solution. Then, the optical density was measured at 450 nm for each sample. All the experiments were performed in triplicate.

### Wound-healing assay

Wound healing assay was done for assessment of cell migration. Cells at a density of 1 × 10^3^ were seeded in 12-well plates and incubated in medium for 12 h. Then artificial wound was created using a 200 μL micropipette tip on the surface of plates. The wound was observed and pictures were taken at indicated time points (0 and 24 h).

### Transwell invasion assay

Transwell chambers (with 8-μm pore size; Sigma-Aldrich, St. Louis, MO, USA) coated with matrigel were used in transwell assay to examine cell invasion. Briefly, 1×10^5^ cells in serum-free RPMI-1640 medium were plated into the upper chamber. RPMI-1640 medium supplemented with 10% FBS was added to the lower chamber. After being cultured for 24 h, cells invaded to the lower chamber were fixed with methanol and stained with crystal violet. The number of invaded cells was counted from five fields, which were randomly selected in the lower chamber.

### Western blot

Firstly, RIPA lysis buffer (Beyotime Biotechnology, China) was utilized to extract total proteins. Protein concentration was then measured using BCA™ Protein Assay Kit (Pierce, Appleton, USA). Subsequently, protein extracts were separated using 10% SDS-PAGE gel and transferred to polyvinylidene fluoride membranes (Millipore, Billerica, MA, USA). The membranes were blocked in 5% skim milk for 2 h at room temperature, and then incubated overnight at 4 °C with diluted primary antibody. Following washed with TBST, the membranes were incubated with secondary antibody for 2 h at room temperature. Finally, the ECL chemiluminescent detection system (Thermo Fisher Scientific, Rochester, NY) was utilized to detect target bands. The primary antibodies used were as follows: anti-PAI1 (13801-1-AP, Proteintech, USA), anti-SERBP1 (14088-1-AP, Proteintech), anti-SIX5 (abcam, UK), anti-GAPDH (60004-1-Ig, Proteintech), anti-USP5 (ab154170, Abcam, Cambridge, MA, USA) and anti-Histone 3 (ab1791, Abcam, Cambridge). The original western blots were shown in Supplemental Material.

### RNA extraction and reverse-transcription quantitative real-time PCR (RT-qPCR)

Total RNA was extracted with Trizol reagent (Takara, Otsu, Japan) following the manufacturers’ instruction. For mRNA expression analysis, reverse transcription was performed using PrimeScript RT reagent kit (TaKaRa, Dalian, China). SYBR Green PCR Kit (Takara) was used to carry out Polymerase chain reaction (PCR). Thermal Cycler Dice Real-Time PCR System (Takara, Japan) was utilized to conduct Real-time PCR. GAPDH was used as endogenous controls. The fold-change of genes was calculated using 2^–ΔΔCt^ method. Primers used in this study were presented as follows: LINC01468: 5’-TTA CTG CCA CAC TCC CGT TC-3’ (Forward) and 5’-CAT GGG CAG ACC ACT CAT GT-3’ (Reverse); SERPINE1: 5’- CCC TCT ACT TCA ACG GCC AG-3’ (Forward) and 5’- GAG CTG GGC ACT CAG AAT GT-3’ (Reverse); SERBP1: 5’-CTG ACC GAC ATC GAA GGT GA-3’ (Forward) and 5’-AAG TGC AAT CCA TGG CTC CG-3’ (Reverse); SIX5: 5’- TGC AAG AGC GAG TCT GAT GG-3’ (Forward) and 5’- GGT CCC TGC CAG GAA TAT GG-3’ (Reverse); GAPDH: 5’-GAA GGT GAA GGT CGG AGT C-3’ (Forward) and 5’-GAA GAT GGT GAT GGG ATT TC-3’ (Reverse).

### Dual-luciferase reporter assay

Wild-type and mutated LINC01468 promoter sequences were cloned into the pGL3 basic luciferase reporter vectors to construct pGL3/LINC01468-WT and pGL3/LINC01468-MUT (site 1)/(site 1&2). Then, HEK 293T, A549 and SPC-A1 cells were co-transfected with the abovementioned constructs and SIX5 overexpression plasmid or the control plasmid with Lipofectamine 2000. Luciferase reporter assay system (Promega, Madison WI, USA) was used to detect luciferase activities at 48 h post transfection.

### RNA pull-down assay

RNA-Protein Pull down Kit (Thermo Fisher Scientific). Briefly, biotin-labeled RNAs were mixed with magnetic beads and then incubated with protein lysate at 4 °C with rotation for 4 h. After washing process (five times), the bound proteins were eluted and detected by western blot.

### RNA-binding protein immunoprecipitation assay (RIP)

RIP assay was conducted with Magna RIP™ RNA-Binding Protein Immunoprecipitation Kit (Millipore, Billerica, MA, USA) following the instructions. Briefly, cells were lysed using RIP lysis buffer, and then cell extract were incubated with magnetic beads, which were coated with SERBP1 antibodies, respectively. Input and normal IgG were used as controls. Immunoprecipitated RNAs were purified by proteinase K and detected by RT-qPCR.

### Chromatin immunoprecipitation (ChIP) assay

SimpleChIP® Enzymatic Chromatin IP Kit (CST, USA) was utilized to conduct ChIP assay according to the manufacturers’ protocol. Firstly, enzymatic digestion was used to break cross-linked chromatin into 200 to 1000 bp fragments. The chromatin was immunoprecipitated using Anti-SIX5 or Anti-IgG (control) at 4 °C overnight with rotation. Subsequently, magnetic beads were added into tube and incubated at 4 °C with rotation for 2 h. Then the immunoprecipitated chromatin was washed with low-salt solution three times and high-salt solution one time. Finally, the immunoprecipitated chromatin was purified and analyzed by RT-qPCR.

### Subcellular fractionation

In order to determine the cellular localization of LINC01468 and SERBP1, NUCLEI EZ PREP NUCLEI ISOLATION KIT (Sigma, USA) was used to isolate nuclear fraction from cytoplasm according to the instructions. Firstly, the cells were washed with ice-cold PBS twice. Then cells were lysed by ice-cold Lysis Solution and harvested by a cell scraper on ice. Subsequently, cell lysate was centrifuged at 10,000 rpm for 30 min at 4 °C, the nuclear fraction was in the precipitate while the cytoplasmic fraction in the supernatant. Finally, the supernatant was carefully removed to a new 1.5 ml EP tube. The precipitate was washed by PBS twice and re-suspended by Nuclei EZ storage buffer. The level of LINC01468 was determined through RT-qPCR while the level of SERBP1 was measured via western blot.

### Fluorescence in situ hybridization (FISH) and immunofluorescence (IF) analysis

For FISH analysis of LINC01468 and SERPINE1, probes of LINC01468, and SERPINE1 were synthesized by Bersinbio Company (Guangzhou, China). Firstly, cell slides were fixed with 4% paraformaldehyde for 20 min. The slides were washed with PBS twice and dehydrated by ethanol. Thereafter, slides were denatured, added with 20 μL hybridization reaction solution (2 μL probes + 18 μL hybridization reaction) and hybridized at 37 °C overnight. After that, the slides were washed by 25% formamide/2 × Saline Sodium Citrate (SSC) twice. For IF analysis of SERBP1, SERBP1 antibody was utilized. Cells after fixation and permeablization were incubated with the primary antibody overnight and then incubated with secondary antibody for 1 h. Finally, the slides were stained with DAPI and subjected to fluorescent signal detection using Zeiss LSM800 confocal laser microscopy (Zeiss, Germany).

### Co-immunoprecipitation (Co-IP) analysis

Then total protein was extracted from cells and incubated with control anti-IgG, anti‑PAI1 antibody overnight at 4 °C. Then the Protein A/G (Santa Cruz Biotechnology, Dallas, TX, USA) was added and incubated for overnight at 4 °C. Immobilized protein complexes were extensively washed with the PBS, re-suspended in loading buffer, and separated on SDS-polyacrylamide gel electrophoresis.

### Animal experiments

Animal experiments were done to investigate the influences of LINC01468 and SERPINE1 on LUAD progression under the approval of Nanjing First Hospital, Nanjing Medical University. Briefly, BALB/c nude mice obtained from Institute of Model Fauna, Nanjing University were casually divided into groups. SPC-A1 cells, respectively, transfected with shCtrl, sh-LINC01468#1 or sh-SERPINE1#1 were subcutaneously injected into mice. Tumor volumes were measured every 3 days since day 7 according to the formula: Volume = (length × width^2^)/2. At day 28, all mice were sacrificed and tumor weight was calculated. Additionally, the tumors were excised and collected for Immunohistochemistry (IHC) assay with anti-PCNA, anti-Ki67, Anti-E-cadherin and Anti-N-cadherin used.

### Statistical analyses

Statistical analyses were carried out by SPSS 20.0 software (SPSS Inc., USA) and data collected from three independent experiments were shown as mean ± SD. The significant difference was analyzed by Student’s *t*-test and ANOVA as needed and presented as *p* < 0.05.

## Results

### LINC01468 is upregulated in LUAD tissues and cell lines

**Fig. 1:**
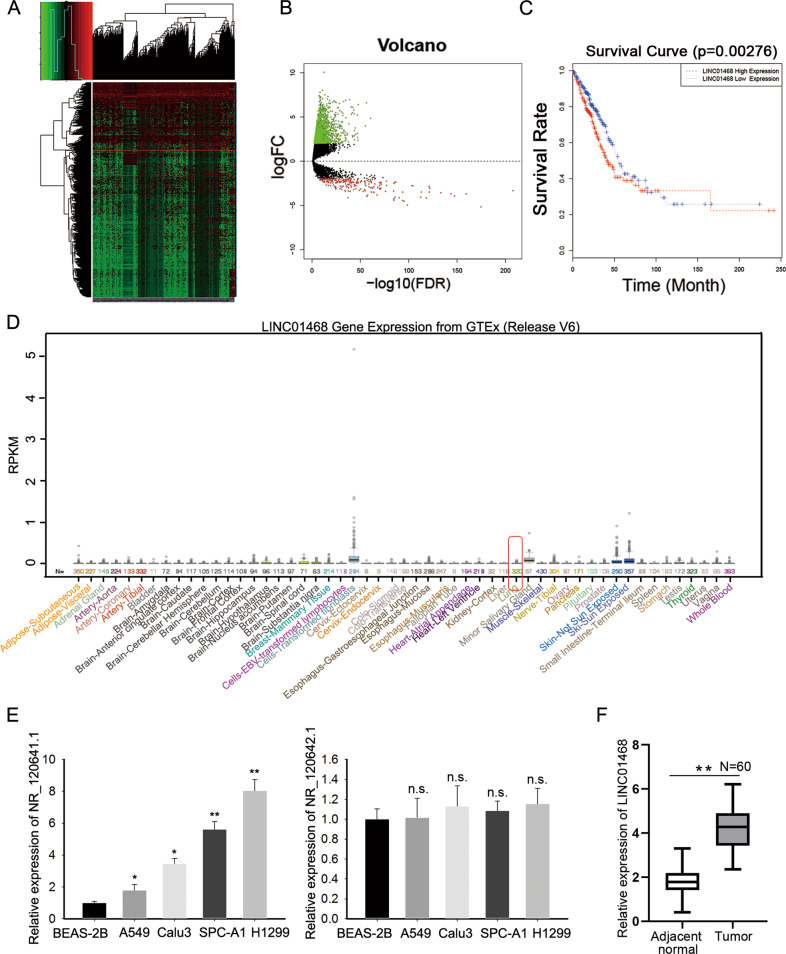
LINC01468 is upregulated in LUAD tissues and cell lines. **A** A heat map was generated to show the lncRNA profiles in TCGA lung cancer samples. **B** A volcano plot recorded the differentially expressed lncRNAs (fold-change > 2.0). **C** Survival curve was plotted to analyze the overall survival of lung cancer patients with high or low LINC01468 expression. **D** LINC01468 expression profile in normal tissues was obtained from UCSC. **E** The expression level of different LINC01468 transcripts in LUAD cell lines and normal cell line BEAS-2B was examined by RT-qPCR. **F** LINC01468 expression was examined in 60 pairs of LUAD tissues and adjacent normal tissues by RT-qPCR. Results were exhibited as the mean ± SD on the basis of three independent experiments. *p < 0.05, **p < 0.01 indicated the statistical significance of experiments data. n.s.: no significance.

To find out the potential functional lncRNAs that played a role in the progression of LUAD, data from The Cancer Genome Atlas (TCGA) database (http://cancergenome.nih.gov/) was analyzed. Differentially expressed lncRNAs in LUAD tissues compared with normal ones were presented in the heatmap and the volcano plot (Fig. 1A, B). LINC01468 was one of the significantly overexpressed genes in LUAD tumor tissues, whose function in LUAD and other malignancies was rarely investigated. Therefore, LINC01468 was chosen as our research target. More importantly, the overall survival (OS) curve based on TCGA data suggested that patients with higher LINC01468 level had shorter survival time (Fig. 1C). In addition, the results from UCSC (http://www.ucsc.edu/) online database demonstrated that low expression of LINC01468 was observed in normal lung tissues (Fig. 1D). So, we chose LINC01468 as our research target. More specifically, by detecting the level of two transcripts (NR_120641.1 and NR_120642.1) of LINC01468 RT-qPCR in four LUAD cell lines (A549, SPC-A1, H1975, and H1299) and BEAS-2B cells, we discovered that only transcript NR_120641.1 displayed aberrantly high expression in LUAD cell lines (Fig. 1E). Therefore, we focused on the transcript NR_120641.1 in our subsequent experiments. Afterwards, LINC01468 expression was examined in 60 pairs of LUAD tissues and adjacent normal tissues. As expected, LINC01468 expressed higher in LUAD tissues (Fig. 1F). Aiming to assess the clinical significance of LINC01468, we evaluated the correlation of LINC01468 expression with clinicopathological characteristics. As manifested in Table 1, LINC01468 expression was correlated with tumor size (*p* < 0.001) and TNM (*p* = 0.019) while there existed no significant correlation between LINC01468 level and other characteristics including age, gender, smoking history, tumor location and differentiation (*p* > 0.05). Taken together, upregulated LINC01468 might serve as a crucial effector in modulating LUAD progression.

**Table 1:** The association between LINC01468 expression and the clinicopathologic characteristics of LUAD patients was demonstrated.

The association between LINC01468 expression and the clinicopathologic characteristics of lung cancer patients.				
Parameters		LINC01468 expression		*p*-value
		Low expression	High expression	
Number		30	30	
Age	<60	9	5	0.360
	≥60	21	25	
Gender	Male	13	20	0.119
	Female	17	10	
Smoking History	No	14	7	0.103
	Yes	16	23	
Tumor location	Left	7	4	0.506
	Right	23	26	
Differentiation	High	14	6	0.054
	Poor	16	24	
Tumor size	≤3	17	4	<0.001***
	>3	13	26	
TNM	I/II	20	10	0.019*
	III/IV	10	20	

***p < 0.001, *p < 0.05.

### LINC01468 promotes cell proliferation and migration in vitro

**Fig. 2:**
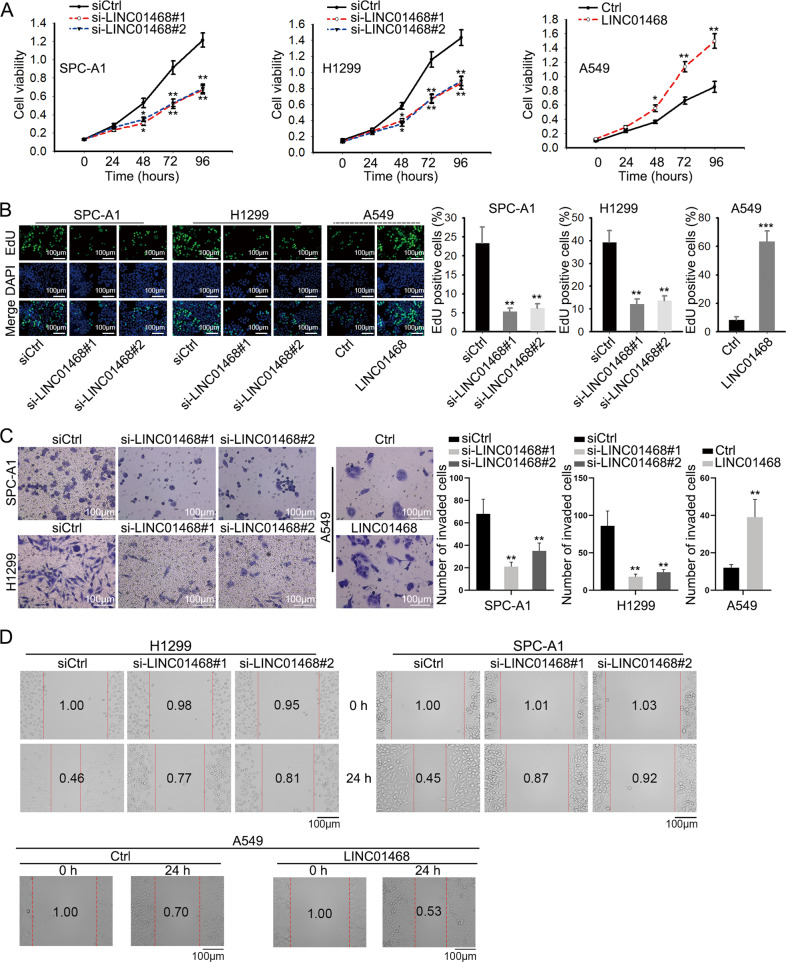
LINC01468 promotes LUAD cell proliferation, migration, and invasion. **A**, **B** CCK-8 and EdU assays were used to analyze the effect of LINC01468 knockdown or overexpression on cell proliferation. **C**, **D** Invasive and migratory ability of LUAD cells with LINC01468 deficiency or overexpression was detected by transwell invasion and wound healing assays. Results were exhibited as the mean ± SD on the basis of three independent experiments. *p < 0.05, **p < 0.01 indicated the statistical significance of experiments data.

To assess the impacts of LINC01468 on LUAD cell behaviors, we knocked down LINC01468 in LUAD cell lines with highest LINC01468 expression (SPC-A1 and H1299) or overexpressed LINC01468 in LUAD cell line with relatively low LINC01468 level (A549), and detected the change in cell proliferation, migration and invasion. LINC01468 knockdown and overexpression efficiencies were preliminarily determined via RT-qPCR (Supplementary Fig. 1A). As illustrated in CCK-8 and EdU assay results, LINC01468 knockdown caused a remarkable inhibition on LUAD cell proliferation, while LINC01468 overexpression induced stronger proliferation ability of LUAD cells (Fig. 2A, B). Next, transwell invasion assay unveiled that the depletion of LINC01468 curbed LUAD cell invasion, and the augment of LINC01468 had opposite effect (Fig. 2C). Wound healing assays further confirmed the promoting role of LINC01468 in modulating LUAD cell migration (Fig. 2D). Animal experiments were done to analyze the function of LINC01468 in vivo, and sh-LINC01468 was used for LINC01468 knockdown (Supplementary Fig. 1B). As was revealed in Supplementary Fig. 1C–E, LINC01468 could suppress tumor growth. IHC experiment was performed to test the expression of proliferation and invasion markers. Increased level of E-cadherin and decreased level of PCNA, Ki67 and N-cadherin were discovered as a result of LINC01468 depletion (Supplementary Fig. 1F). To sum up, LINC01468 promoted LUAD cell proliferation, migration and invasion in vitro and accelerated tumor growth in vivo.

### SIX5 contributes to LINC01468 upregulation

**Fig. 3:**
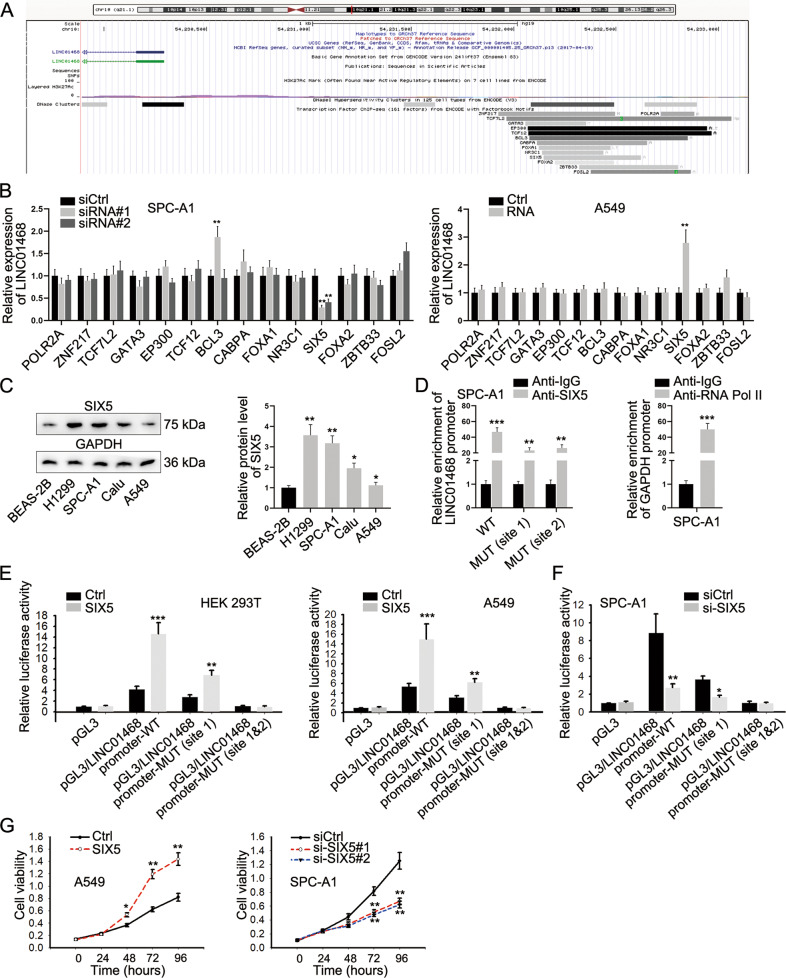
SIX5 contributes to LINC01468 upregulation in LUAD cells. **A** UCSC was applied for prediction of candidate transcription factors for LINC01468. **B** The alterations in LINC01468 after knocking down or overexpressing candidate transcription factors were assessed via RT-qPCR, respectively. **C** Expression level of SIX5 in LUAD cell lines and normal cell line was measured via western blot analysis. **D** The affinity of SIX5 and LINC01468 promoter was determined by ChIP assay. **E**, **F** The impact of SIX5 on the transcriptional activity of LINC01468 was determined by luciferase reporter assay and the exact binding site of SIX5 on LINC01468 promoter was verified. **G** Proliferative ability of cells with SIX5 overexpression or silence was assessed by CCK-8 assay. Results were exhibited as the mean ± SD on the basis of three independent experiments. *p < 0.05, **p < 0.01, ***p < 0.001 indicated the statistical significance of experiments data.

To investigate the regulatory mechanism underlying LINC01468 upregulation in LUAD cell lines, we predicted the transcription factors of LINC01468 on UCSC (http://genome.ucsc.edu/) (Fig. 3A). After knocking down the candidate transcription factors, respectively (Supplementary Fig. 1G and Supplementary file 1), it was discovered that only SIX homeobox 5 (SIX5) could positively modulate LINC01468 expression through RT-qPCR analysis (Fig. 3B). Western blot results indicated that SIX5 was also upregulated in LUAD cell lines (Fig. 3C). Then, the sequence of LINC01468 promoter was obtained and displayed in Supplementary file 2, and the binding sequence to SIX5 was marked in red. On this basis, ChIP assay results showed significantly high enrichment of wild-type LINC01468 promoter in Anti-SIX5, and when LINC01468 promoter was mutated in site 1 or site 2, the enrichment was both partly decreased, indicating SIX5 bound to LINC01468 at both site 1 and site 2 (Fig. 3D). Besides, the luciferase activity of pGL3/LINC01468 promoter-WT and pGL3/LINC01468 promoter-MUT (site 1) was increased under SIX5 overexpression and decreased with SIX5 silence, and that of pGL3/LINC01468 promoter-MUT (site 1&2) was hardly changed under same conditions (Fig. 3E, F). Additionally, SIX5 overexpression was verified to strengthen the proliferative, migratory and invasive abilities of LUAD cells while SIX5 depletion led to the opposite consequences (Fig. 3G and Supplementary Fig. 1H, I), indicating the oncogenic property of SIX5. Collectively, SIX5 accelerated LINC01468 transcription to induce LINC01468 upregulation and upregulation of LINC01468 could result in exacerbated LUAD cell proliferation, migration and invasion.

### LINC01468 binds with SERBP1 in cytoplasm

**Fig. 4:**
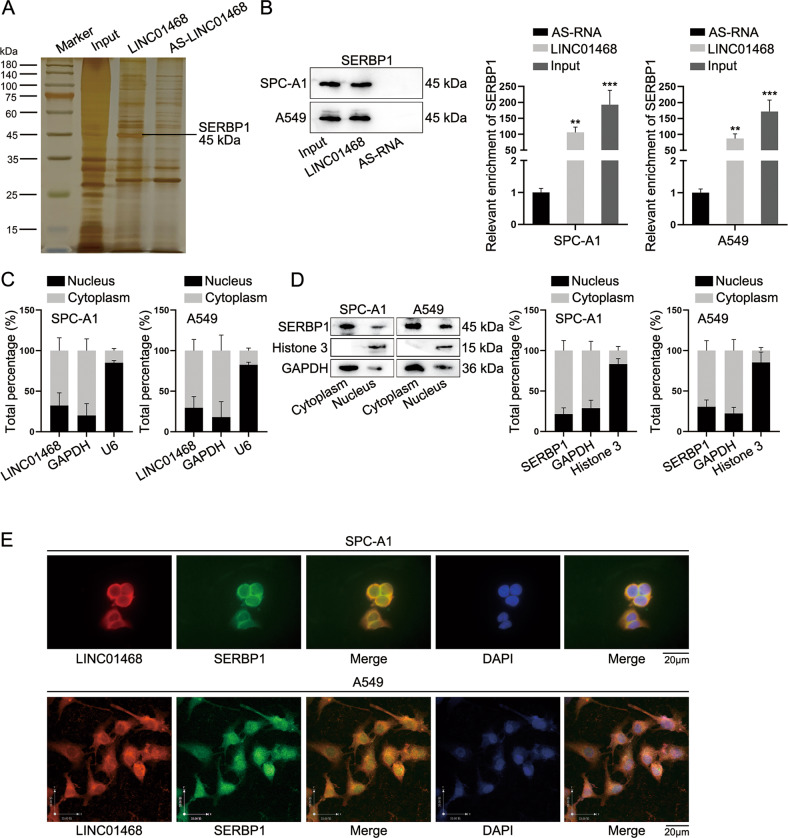
LINC01468 binds with SERBP1 in cytoplasm. **A** RNA pull-down assay with mass-spectrometry was performed to obtain LINC01468-binding proteins. **B** After RNA pull-down assay, western blot analysis was done for measurement of SERBP1 level pulled down by indicated probes. **C**, **D** After subcellular fractionation, RT-qPCR and western blot analyses were, respectively, done to examine the accumulation of LINC01468 and SERBP1 in cytoplasm and nucleus of LUAD cell lines. **E** FISH-IF was applied to determine the co-localization of LINC01468 and SERBP1 in LUAD cells. Results were exhibited as the mean ± SD on the basis of three independent experiments. **p < 0.01, ***p < 0.001 indicated the statistical significance of experiments data.

To identify the detailed mechanism by which LINC01468 functioned in LUAD cells, biotin RNA-protein pull-down assay followed by mass-spectrometry was carried out to recognize potential binding proteins with LINC01468 (Fig. 4A). From the result, SERBP1 (SERPINE1 mRNA binding protein 1), which was previously discovered to be differentially expressed in lung cancer [29], was found to have relatively high potential to bind with LINC01468. The binding of SERBP1 and LINC01468 was further confirmed by RNA pull-down assay, the result of which verified that LINC01468 biotin probes could largely pull down SERBP1 (Fig. 4B). Additionally, subcellular fractionation assay followed with RT-qPCR and western blot analyses revealed that LINC01468 and SERBP1 were mainly accumulated in the cytoplasm of SPC-A1 and A549 cell lines (Fig. 4C, D). Results of FISH-IF indicated that LINC01468 and SERBP1 were co-localized in cytoplasm (Fig. 4E). Subsequently, functional assays were carried out to determine the function of SERBP1 in the regulation of LUAD cell behaviors. According to Supplementary Fig. 2A–E, LUAD cell proliferation, migration and invasion capabilities were all repressed after si-SERBP1 depletion but strengthened as a result of SERBP1 overexpression. All these findings indicated that LINC01468 could interact with SERBP1 in the cytoplasm of LUAD cell lines and SERBP1 could exacerbate LUAD cell malignant behaviors.

### LINC01468 stabilizes SERPINE1 mRNA by binding to SERBP1

**Fig. 5:**
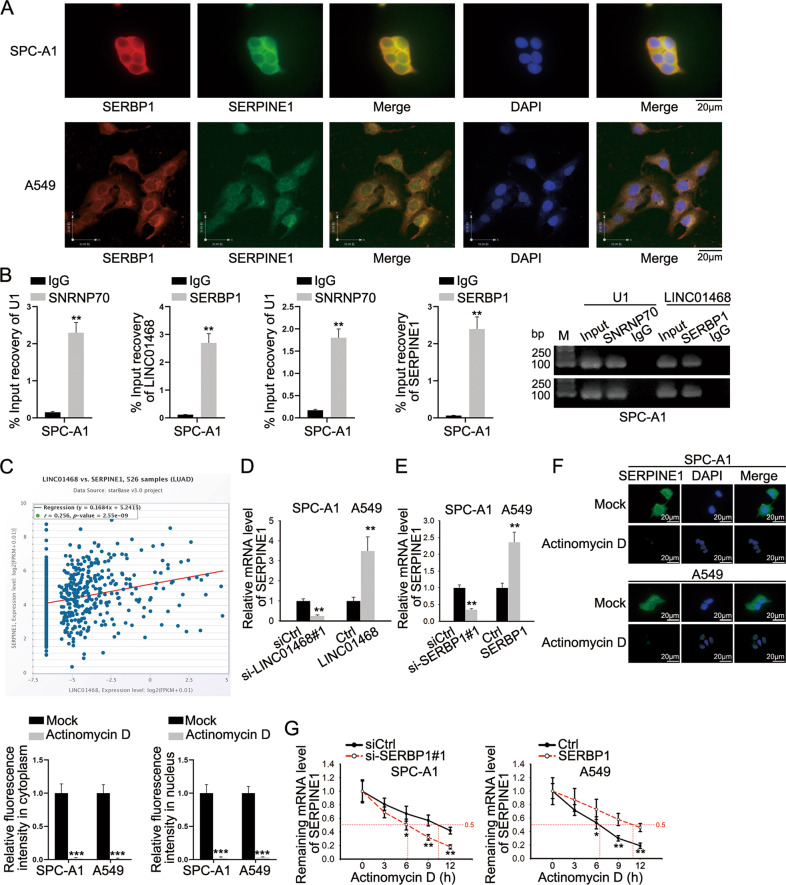
LINC01468 stabilizes SERPINE1 mRNA by binding to SERBP1. **A** FISH-IF was applied to determine the cellular distribution of SERBP1 and SERPINE1. **B** RIP assay was done for assessment of the combination between SERBP1 and LINC01468/SERPINE1. **C** The correlation between LINC01468 and SERPINE1 expression in LUAD was predicted on starBase. **D**, **E** The mRNA level of SERPINE1 was measured via RT-qPCR in cells with silence or overexpression of LINC01468 or SERBP1. **F** The changes in cytoplasmic and nuclear accumulation of SERPINE1 under Actinomycin D addition were detected via FISH. **G** SERPINE1 mRNA stability was assessed in Actinomycin D-treated cells under the influence of SERBP1 deficiency or overexpression. Results were exhibited as the mean ± SD on the basis of three independent experiments. *p < 0.05, **p < 0.01 indicated the statistical significance of experiments data.

SERBP1 is the RBP which can regulate the mRNA stability of SERPINE1. More importantly, SERPINE1 has been reported as a facilitator of tumor growth and metastasis [30–32]. Since LINC01468 could bind with SERBP1 in LUAD cells, we speculated that it might have an effect on the stability of SERPINE1 mRNA. The co-existence of SERPINE1 and SERBP1 in LUAD cell cytoplasm was first identified via FISH-IF assays (Fig. 5A). Then, RIP assay verified that anti-SERBP1 immunoprecipitated both LINC01468 and SERPINE1 (Fig. 5B and Supplementary Fig. 2F). The positive correlation between LINC01468 and SERPINE1 in LUAD tissues was predicted on starBase (https://starbase.sysu.edu.cn/index.php) (Fig. 5C). Furthermore, we found that LINC01468 depletion resulted in decreased mRNA level of SERPINE1 while LINC01468 overexpression augmented SERPINE1 expression (Fig. 5D). SERBP1 inhibition could also lead to SERPINE1 mRNA decrease while SERBP1 augment could reduce increase in mRNA level of SERPINE1 (Fig. 5E). The influence of SERBP1 on SERPINE1 mRNA stability was evaluated with addition of Actinomycin D (inhibitor of mRNA synthesis). Before testing the mRNA stability, FISH assay was done in advance to detect SERPINE1 expression in different cellular parts with or without Actinomycin D treatment. It was evidenced that SERPINE1 level in both cell cytoplasm and nucleus declined after Actinomycin D addition (Fig. 5F). Therefore, the influence of nascent SERPINE1 was excluded. As shown in Fig. 5G, the half-life of SERPINE1 was obviously shortened after silencing SERBP1, while the opposite result was observed after overexpression of SERBP1. In a word, LINC01468 stabilized SERPINE1 mRNA through binding with SERBP1.

### LINC01468 recruits USP5 to facilitate PAI1 protein deubiquitylation

**Fig. 6:**
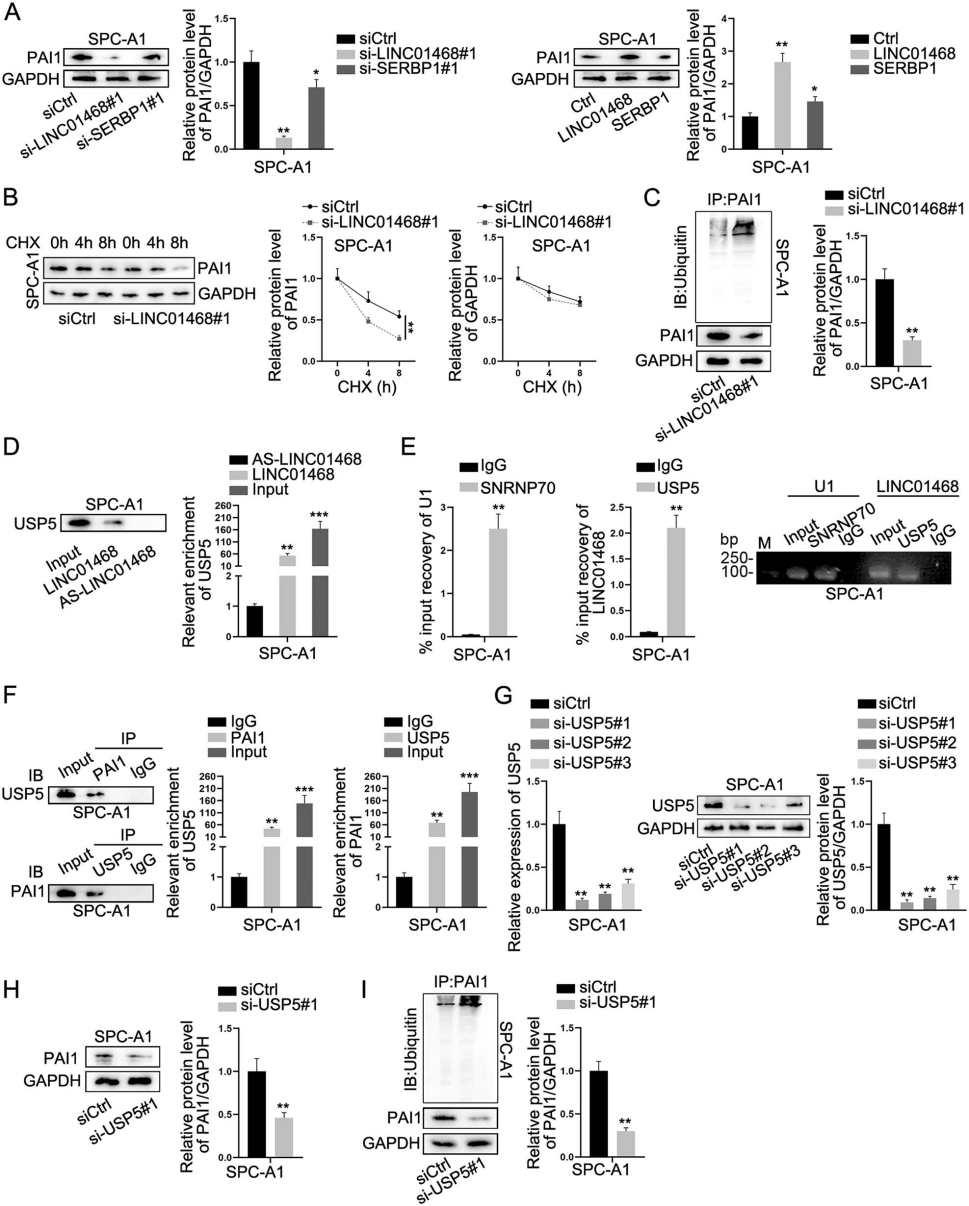
LINC01468 impedes PAI1 protein degradation via interaction with USP5. **A** Protein levels of PAI1 in response to the knockdown or overexpression of LINC01468 or SERBP1 were measured via western blot. **B** After CHX treatment, western blot analysis was done to measure protein levels of PAI1 in cells with or without LINC01468 knockdown. **C** IP-WB was done for assessment of PAI1 ubiquitination under LINC01468 silence. **D** After RNA pull-down assay, western blot was done to detect USP5 level pulled down by indicated probes. **E** The binding correlation between LINC01468 and USP5 was verified via RIP assay. **F** Co-IP assay was applied for evaluation of the interaction between USP5 and PAI1. **G** The changes in USP5 mRNA and protein levels in cells transfected with si-USP5 were assessed via RT-qPCR and western blot. **H** The influence of USP5 silence on PAI1 level was evaluated via western blot. **I** IP-WB was applied for assessment of PAI1 ubiquitination under the influence of USP5 depletion. Results were exhibited as the mean ± SD on the basis of three independent experiments. *p < 0.05, **p < 0.01^,^ ***p < 0.001 indicated the statistical significance of experiments data.

For further exploration of the LINC01468 regulatory mechanism, we conducted western blot analysis to detect the expression of Plasminogen Activator Inhibitor-1 (PAI1) (corresponding protein of SERPINE1 mRNA). Interesting, while knockdown or overexpression of LINC01468 could substantially decrease or increase the protein level of PAI1, the depletion or augment of SERBP1 had less obvious influence on modulation of PAI1 level (Fig. 6A), implying that LINC01468 might affect PAI1 level via regulatory mechanism. Therefore, we treated siCtrl or si-LINC01468 transfected LUAD cells with CHX (protein synthesis inhibitor) and detected PAI1 level at different time points. From the results, we discovered PAI1 degraded more quickly when LINC01468 was depleted (Fig. 6B). Ubiquitylation is the most common protein regulation way at post-translational level [33]. To ravel out whether LINC01468 could affect PAI1 ubiquitylation to affect PAI1 degradation, we conducted IP-WB and ensured LINC01468 depletion had promoting effect on PAI1 ubiquitylation while the protein level of PAI1 was decreased (Fig. 6C). Moreover, we discovered that USP5, one of the RBPs of LINC01468 previously determined in the RNA pull-down assay and mass-spectrometry analyses, could mediate the deubiquitylation of proteins [34]. Thus, we hypothesized that LINC01468 might recruit USP5 to modulate the deubiquitylation of PAI1. RNA pull-down assay and RIP assay proved the cohesion between LINC01468 and USP5 (Fig. 6D, E), and subsequent Co-IP assay verified the binding between USP5 and PAI1 (Fig. 6F). Next, we ascertained that PAI1 expression was decreased with the knockdown of USP5 and increased in response to USP5 overexpression (Fig. 6G, H). More specifically, IP-WB evidenced that after si-USP5 transfection, ubiquitylation of PAI1 was enhanced and PAI1 level was lessened (Fig. 6I). In summary, LINC01468 recruited USP5 to deubiquitylate PAI1, thus restraining the degradation of PAI1.

### LINC01468 promotes cell proliferation, migration, and invasion through SERPINE1

**Fig. 7:**
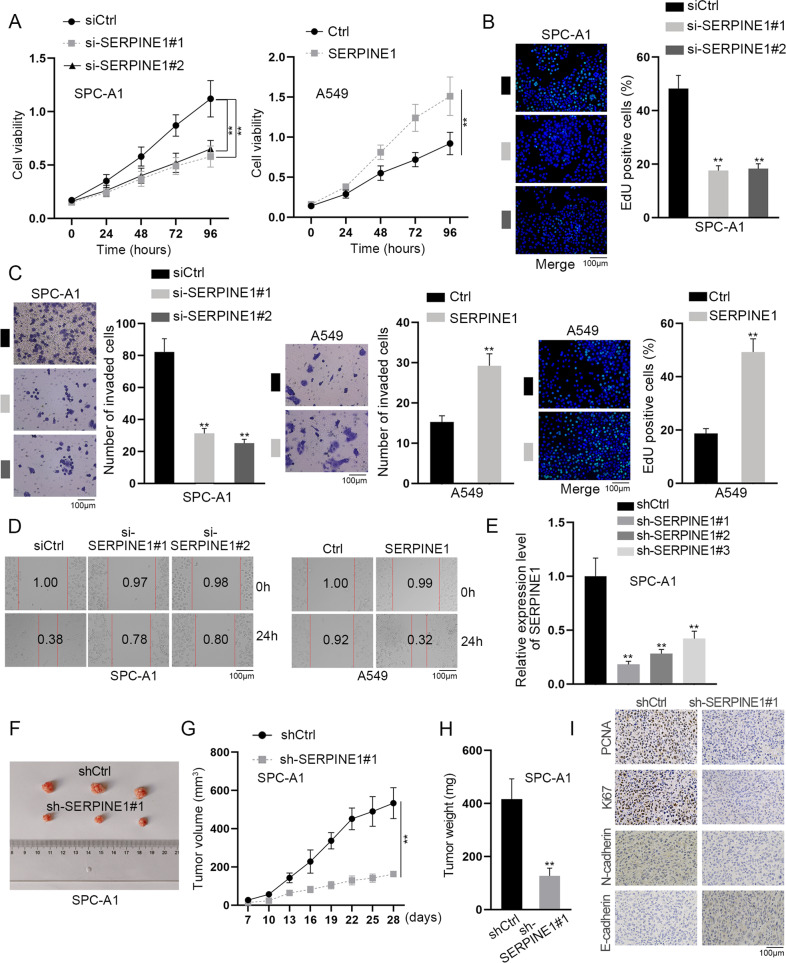
SERPINE1 promotes LUAD cell proliferation, migration and invasion in vitro and facilitates tumor growth in vivo. **A**, **B** Proliferative ability of SPC-A1 and A549 cells was examined via CCK-8 and EdU assays after indicated transfections. **C**, **D** Cell invasion and migration were analyzed through implementation of transwell assay and wound healing assay after transfection of indicated plasmids. **E** RT-qPCR was done to determine sh-SERPINE1 knockdown efficiency in SPC-A1 cells. **F**–**H** Animal experiments were done to evaluate SERPINE1 impacts on tumor growth. Representative images of tumors were displayed, and tumor volume and weights were also analyzed. **I** IHC was done to evaluate PCNA, Ki67, N-cadherin, and E-cadherin changes under the influence of SERPINE1 silence. Results were exhibited as the mean ± SD on the basis of three independent experiments. **p < 0.01 indicated the statistical significance of experiments data.

Based on all findings above, we hypothesized that LINC01468 promoted LUAD cell proliferation, migration and invasion via regulation on SERPINE1. To verify our hypothesis, we first carried out in vitro and in vivo assays to determine the function of SERPINE1 in LUAD. CCK-8, transwell invasion and wound healing assays verified that SERPINE1 exerted promoting effects on LUAD cell malignant behaviors in vitro (Supplementary Fig. 2G and Fig. 7A–D), while animal experiments proved SERPINE1 could result in exacerbated tumor growth in vivo (Fig. 7E–I). Then, rescue experiments were implemented with LUAD cells, respectively, transfected with siCtrl, si-LINC01468#1, si-LINC01468#1+Ctrl and si-LINC01468#1 + SERPINE1. After analyzing the results displayed in Supplementary Fig. 3A–D, we noticed that the repressed cell proliferation, migration and invasion due to LINC01468 silence could be rescued by SERPINE1 upregulation while SERPINE1 depletion could countervail the promoting influences of LINC01468 overexpression on LUAD cell proliferation, migration and invasion. Taken together, LINC01468 affected LUAD cell proliferation, migration and invasion via upregulating SERPINE1.

## Discussion

Recently, emerging evidence has proved that lncRNAs play crucial regulatory roles in many diseases due to their involvement in various physiological and pathological processes, including carcinogenesis, therefore becoming potential diagnostic or therapeutic targets for multiple malignancies [25, 35]. LncRNAs function in tumorigenesis mainly owing to their regulation on cellular processes, including cell proliferation, differentiation, apoptosis, migration, invasion and so on [36–40]. For instance, Wu, D. et al. demonstrated that upregulation of lncRNA RAB1A-2 induces FGF1 expression worsening lung cancer prognosis [41]. Ma et al. [42] suggested that lncRNA GCAWKR promotes gastric cancer development by scaffolding the chromatin modification factors WDR5 and KAT2A. In the current study, we found that LINC01468 was highly expressed in lung cancer tissues using online tools and confirmed this result in LUAD tissues and cell lines. Besides, functional assays revealed that LINC01468 played an oncogenic role in vitro.

SIX5 belongs to the SIX family (SIX1-6) that are a class of evolutionarily conserved transcription factors that involves in cell adhesion, cell division, cell death and cell movement [43]. Recently, SIX5 has also been identified to play a key role in cancers. For example, SIX5 is potentially a molecular trigger of pathogenesis in borderline epithelial ovarian tumors [44]. Consistently, we uncovered that SIX5 was the transcription factor of LINC01468 and positively modulated LINC01468 transcription.

To date, it is well known that lncRNAs function as signals, decoys, guides, and scaffolds to modulate the downstream targets at different levels, including transcriptional, post-transcriptional, translational, post-translational and epigenetic level [45–47]. According to the cellular localization of LINC01468 in LUAD cell lines, we explored its post-transcriptional regulatory functions. Previous studies have revealed that lncRNAs can interact with RBP to regulate the mRNA stability of their downstream genes [48–50]. In this study, SERBP1 was found to interact with LINC01468. Importantly, SERBP1 enhanced the mRNA stability of SERPINE1. Meanwhile, LINC01468 also interacted with USP5, which deubiquitylated PAI1 to maintain PAI1 protein stability. PAI1 is a serine protease inhibitor (serpin) that has been proved to have a potentially pro-oncogenic role in cancer via regulating angiogenesis and tumor cell survival [51]. Chen et al. [52] have revealed that silencing of plasminogen activator inhibitor-1 suppresses colorectal cancer progression and liver metastasis. Overexpression of PAI-1 contributes to angiogenesis, metastasis and poor prognosis [53]. At present, we illustrated the positive regulation of LINC01468 on PAI1 via recruiting USP5.

In conclusion, our study first unveiled the carcinogenic role of LINC01468 in LUAD. Mechanically, we unveiled that the upregulation of LINC01468 was attributed to the transcriptional activation of SIX5. Moreover, LINC01468 maintained SERPINE1 mRNA stability by recruiting SERBP1 while suppressing PAI1 protein degradation by recruiting USP5. Other potential mechanisms including LINC01468 influences on SERPINE1 transcription will be further investigated in the future study. However, a new insight into finding potential effective targets for the treatment of LUAD could still be provided.

## Supplementary information

Supplementary figures

Supplementary file 1

Supplementary file 2

Supplemental Material

Supplemental Material

aj-checklist

## Data Availability

The data are available from the corresponding author upon reasonable request.
