# Supplementary material for: SIX5-activated LINC01468 promotes lung adenocarcinoma progression by recruiting SERBP1 to regulate SERPINE1 mRNA stability and recruiting USP5 to facilitate PAI1 protein deubiquitylation
Source: Cell Death Dis. 2022 Apr 6;13(4):312. doi: 10.1038/s41419-022-04717-9 (PMC8987051; doi:10.1038/s41419-022-04717-9)

**Supplementary Figure 1** (A) LINC01468 knockdown and overexpression efficiencies were examined via RT-qPCR. (B) RT-qPCR detected LINC01468 expression in SPC-A1 cells transfected with sh-LINC01468. (C-E) Animal experiments were done to evaluate LINC01468 impacts on tumor growth. Representative images of tumors were displayed, and tumor volume and weights were also analyzed. (F) IHC was done to evaluate PCNA, Ki67, N-cadherin and E-cadherin changes under LINC01468 depletion. (G) SIX5 knockdown and overexpression efficiencies were examined via RT-qPCR. (H-I) The migratory and invasive abilities of SIX5-depleted/overexpressed cells were evaluated via transwell invasion and wound healing assays. Results were exhibited as the mean ± SD on the basis of 3 independent experiments. ^**^P < 0.01 indicated the statistical significance of experiments data.


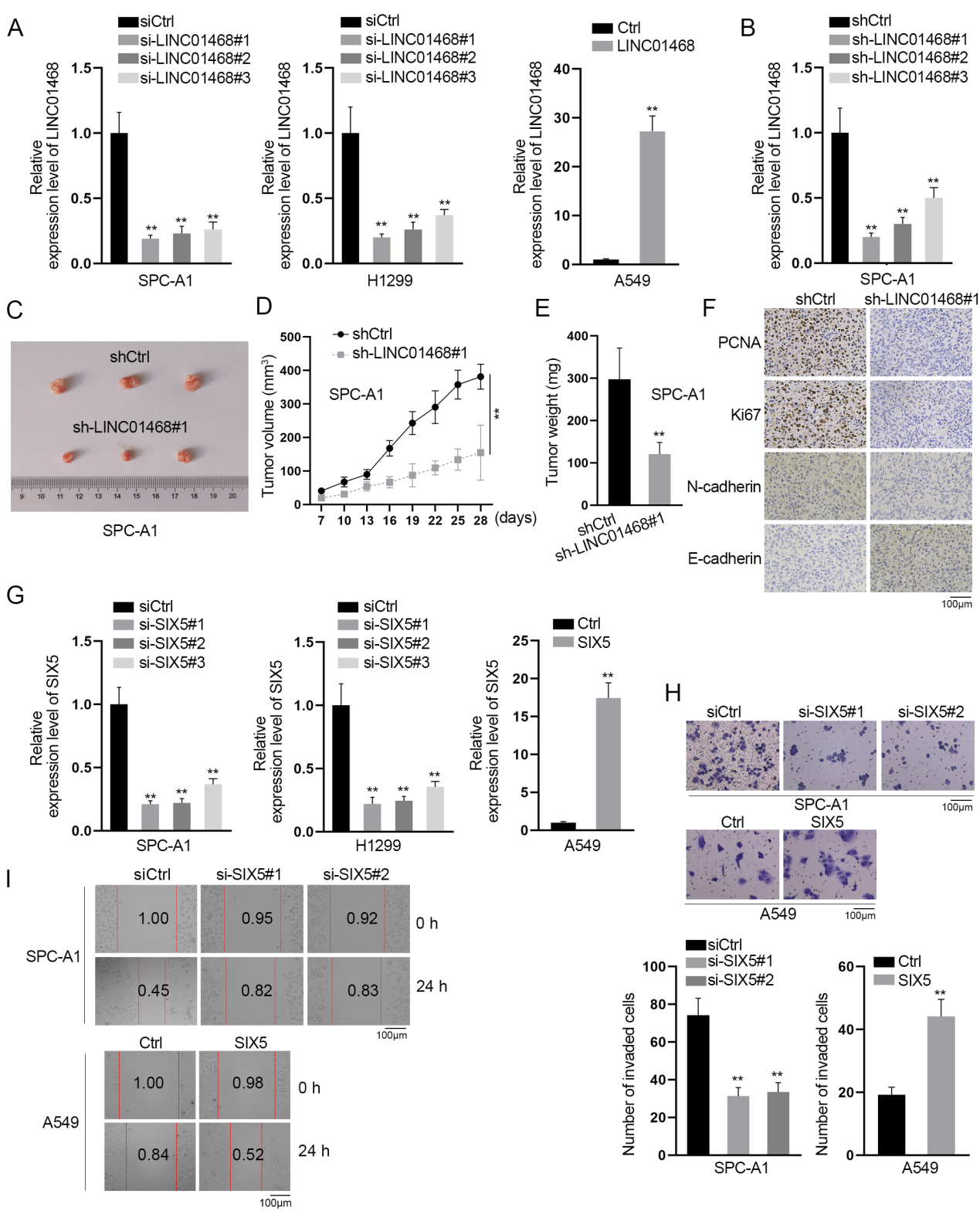


**Supplementary Figure 2** (A) SERBP1 knockdown and overexpression efficiencies were examined via RT-qPCR. (B-E) CCK-8, EdU, transwell and wound healing assays were done to assess the proliferative, invasive and migratory abilities of LUAD cells with SERBP1 silence or overexpression. (F) RIP assay was done for assessment of the combination between SERBP1 and LINC01468/SERPINE1. (G) SERPINE1 knockdown and overexpression efficiencies were determined via RT-qPCR. Results were exhibited as the mean ± SD on the basis of 3 independent experiments. ^**^P < 0.01 indicated the statistical significance of experiments data.


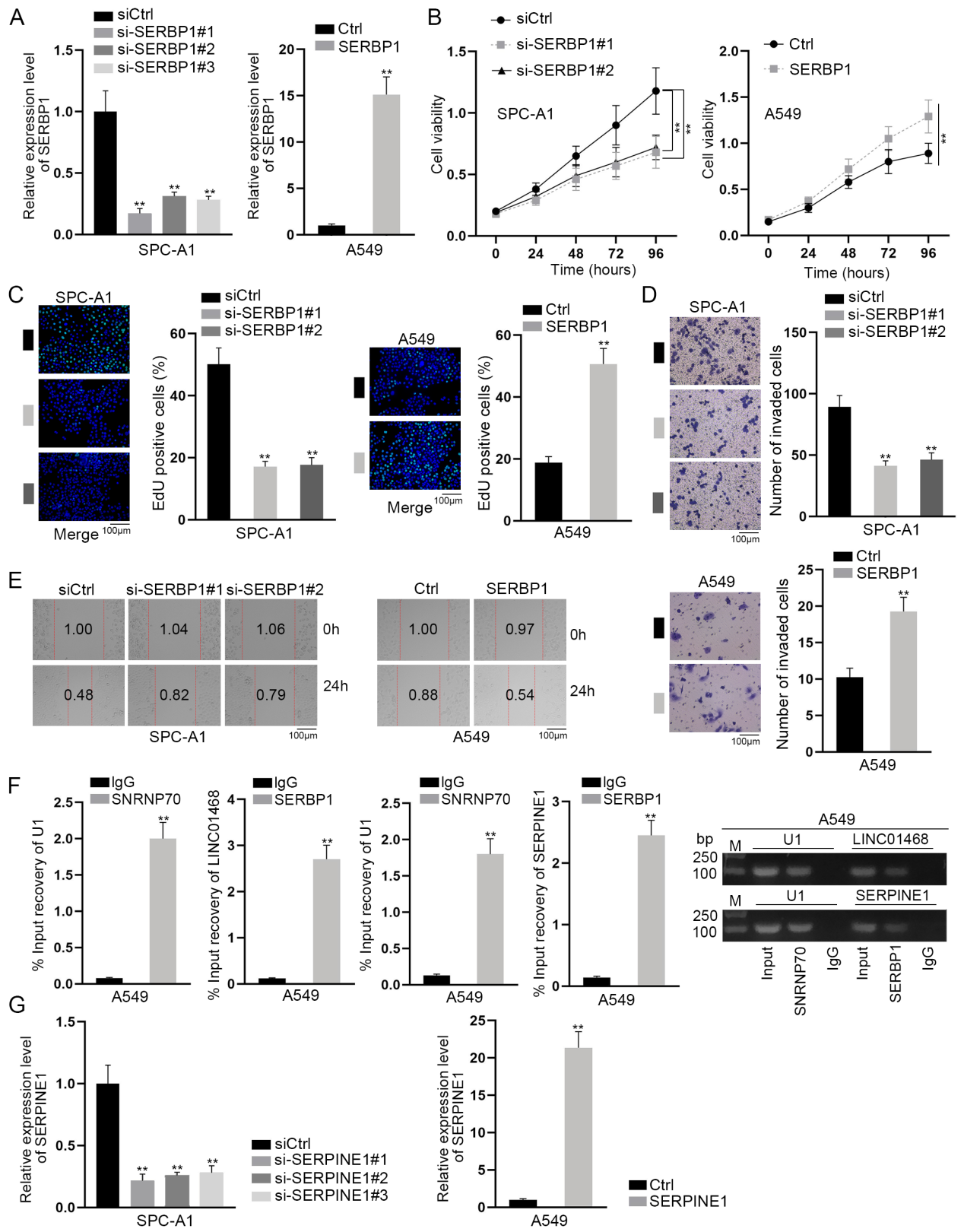


**Supplementary Figure 3** (A-D) The validity of LINC01468/SERPINE1 in modulating LUAD cell malignant behaviors was testified via functional assays including CCK-8, EdU, transwell and wound healing assays. Results were exhibited as the mean ± SD on the basis of 3 independent experiments. ^**^P < 0.01 indicated the statistical significance of experiments data.


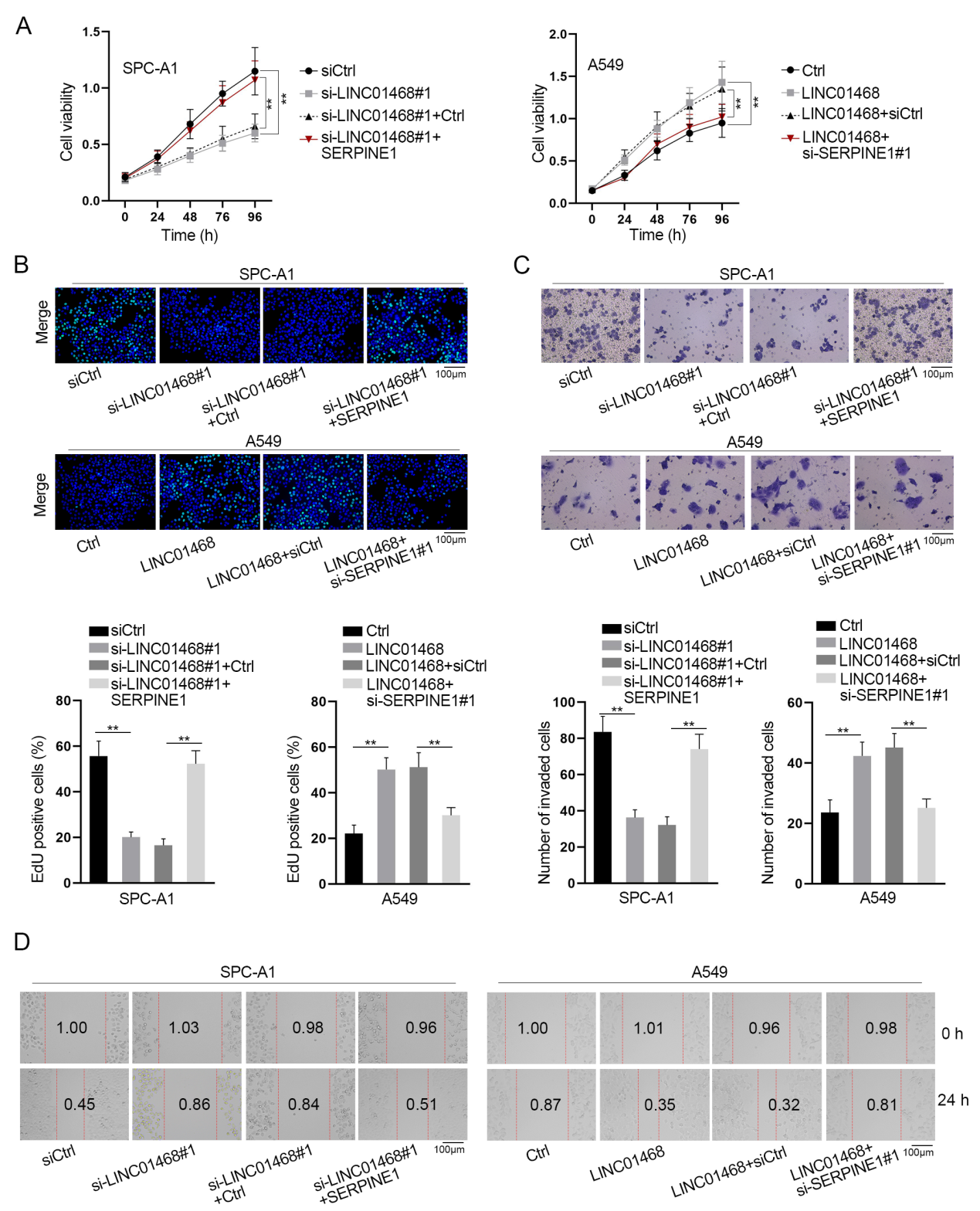

Supplement: Supplementary file 1 — Supplementary figures [file 41419_2022_4717_MOESM1_ESM.docx]
