# Supplementary material for: SIX5-activated LINC01468 promotes lung adenocarcinoma progression by recruiting SERBP1 to regulate SERPINE1 mRNA stability and recruiting USP5 to facilitate PAI1 protein deubiquitylation
Source: Cell Death Dis. 2022 Apr 6;13(4):312. doi: 10.1038/s41419-022-04717-9 (PMC8987051; doi:10.1038/s41419-022-04717-9)

**Supplementary file 1 The knockdown and overexpression efficiencies of 13 predicted transcription factors of LINC01468.**

(A-B) The knockdown and overexpression efficiencies of 13 predicted transcription factors of LINC01468 were detected via RT-qPCR. Results were exhibited as the mean ± SD on the basis of 3 independent experiments. ^**^P < 0.01 indicated the statistical significance of experiments data.


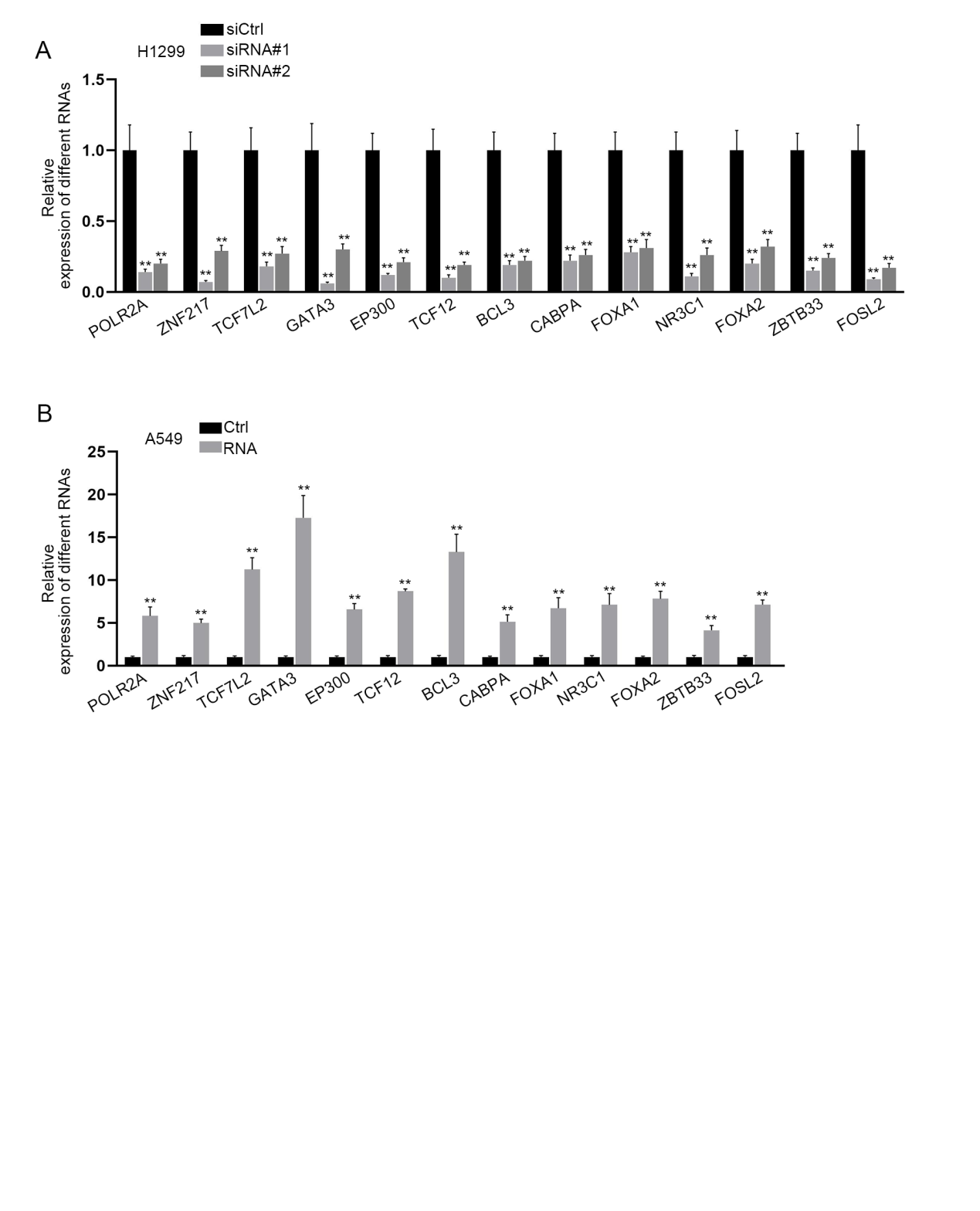

Supplement: Supplementary file 2 — Supplementary file 1 [file 41419_2022_4717_MOESM2_ESM.docx]
