# Supplementary material for: SIX5-activated LINC01468 promotes lung adenocarcinoma progression by recruiting SERBP1 to regulate SERPINE1 mRNA stability and recruiting USP5 to facilitate PAI1 protein deubiquitylation
Source: Cell Death Dis. 2022 Apr 6;13(4):312. doi: 10.1038/s41419-022-04717-9 (PMC8987051; doi:10.1038/s41419-022-04717-9)
Supplement: Supplementary file 3 — Supplementary file 2 [file 41419_2022_4717_MOESM3_ESM.docx]

**Supplementary file 2** Putative binding sites of SIX5 on LINC01468 promoter were provided and marked in red.

>hg19_ncbiRefSeqCurated_NR_120641.1 range=chr10:54210537-54232293 5'pad=0 3'pad=0 strand=-repeatMasking=none

GAATGTTGTTTTCTTAATTTTCTGTTTGTTTGACCTAAGGGCACTAACCGAACTGAGCAAAGGCTTCCAGGAGATGGCTTGGGACAAGAAAAGCAGGTTTCTTTGTTTCTCATGTACAGAGACACATGTCAATCAACCATTGCCTCAGTTAAACATTTAACATCCATGTTAAAAAGAATATCCATTTCACATCCGTCGGTCTGTTACTGGTCTGAAAAGGTGCAGGTTTTGGAAAAATATTTACCATGAGGTCCATCTATGATCTTCAATATGTTCCCCACAGCGAATGATCTCTACTTTCTCTTCAGCTACAATTCCGTGAAAGAATATAATATAGCAGACAATTATAGCAGGGCAAGAGGATTGTTTCATTTACCACCACCAGTAAAGCCAGATTGCAGGGAGGATCAAAGACATTACCTCTTACCAAATCATAATTTTCAGTTATCGGAAATGTTTTTAATTCTGGTAAAAATAATACCAATGACTAATGATTATTTCATGAAAATGCCTAATACTTTTGTTTATTGTGGAGAATTATGATGCTTAATATAAAACTTCTGTGACTTCCAAAGAATAGAGAGAGGGGATTAGGGAAGGGAAGGATGAATGGATGGCTGGAGGTTGAGGCACAACAAATGGGGAAGAGTATGAGTGCCAGTTTTTCCAAGAAGTAATATGTGACTATTAAAAGGCAGGAAGTCACCTAGATTCTTCCAAGTCTTCTGCCAGCTTTTCCCAAGGCTTTCTCACCCCAATAGCAATTTAAATAAGCAATGGAGAAATTGAACCACACTACTGTCATGACCCAGCCTTGGTAAATTCATTTCTAACACAGACCGACAACTCATTTCTCCAGAAATGAGTTTATTTGGATTGATTGCTTCCAAGTAGAGAAATAGCCTCACTTTAAGAAAGCCCTTATTTTTGTTTACAGTTGTTAAGGTCATACATGCAAACACTGTAGGACTTTGTTTTTATTTTTGTTTGATTTGGAGACAAGGTCTCTCTCTGTTACCCAGGCTGGAGTGCAATGGAATATGCATAGCTTACTACAGCCTCTATTCTTGGGCTCAAGCAATCCACCTACCTCAGCTTCCCAAGTAGATAGGACTACACGTGCACCACCATGCTCTGCTAATTAAAAAAACAAAATCTGTAGAGATGGAGCTTTGCTGTGTTACCCAATCTGGTCTCAAATTCCTGGCCTCAAGTAGTCCTCCTGCCTCAGCCTCACAAAGCACTAGGATTACAGGCGTGAGCCATTGTGCCTGGCCTTACTGTAGAACTTTATAATTCATTATCTTCTTTACCCAGGTGTACATAGTATTAGTGAAATAAAATACATTAAATTAAAGCCATTTTTAATTTTTTGGTATACTATGGAATTAGGCATATTTGAAATTACTTCTCATATGACATTGTAAACATAGCCAAATTATTATCATTTTCTTCCTGTAGCAATAAGTCTGAACATAAAATCTTCCATTTTCTTATACAAAATGAAGCTTTAAAAAATCAAGAGCATTTAATTTGTTTCATATATAGGCCTTCTCAACTGCGATCTTGCCCCAGAGAAGAGAAAAAACTACACAGAAACCTTAAGCATTAAGACACATTATTGTAACTAAGAAAGAGCATAACGTTAATAGTGGCTTTTTCTAAGGTGTAAAATAATGAATTTTTAAGATACTTTTCCTTATTTTTCATATTTTGTATATTAAATATATACCACTTCTATCATTTAAAATATCTATTATGTTTTTAAAATAAAGGCTTTCTTTCCGCTTTCTTTCAGGCCAGAGGTAAAAGAAACAGAGAAGAAGAGATAATAATTCTAACAGAAATATTACAGCCTTTACAACTTGCATGCATACAGTTATACATTAAGAGACTGAAAAAGGCAGTTTCTTTTACTGCTAATTCTAGTAGTGATGAAACTCAGGCTCAAGTATTCAGGTGACTTGTTTGAGGTCACACAGTGCATAAATGGTTGCACCGAACTTTTGTCATAGAGGCTAGTCCATTTTATGTCAACGGGGCTTAAATAAGATCTGTGCCAGCAACTGAATTACACTAATGAGTGAGTAATTACAAAAG
