# Supplementary figures and images for: SIX5-activated LINC01468 promotes lung adenocarcinoma progression by recruiting SERBP1 to regulate SERPINE1 mRNA stability and recruiting USP5 to facilitate PAI1 protein deubiquitylation
Source: Cell Death Dis. 2022 Apr 6;13(4):312. doi: 10.1038/s41419-022-04717-9 (PMC8987051; doi:10.1038/s41419-022-04717-9)

**Supplemental Material** Full and uncropped western blots.


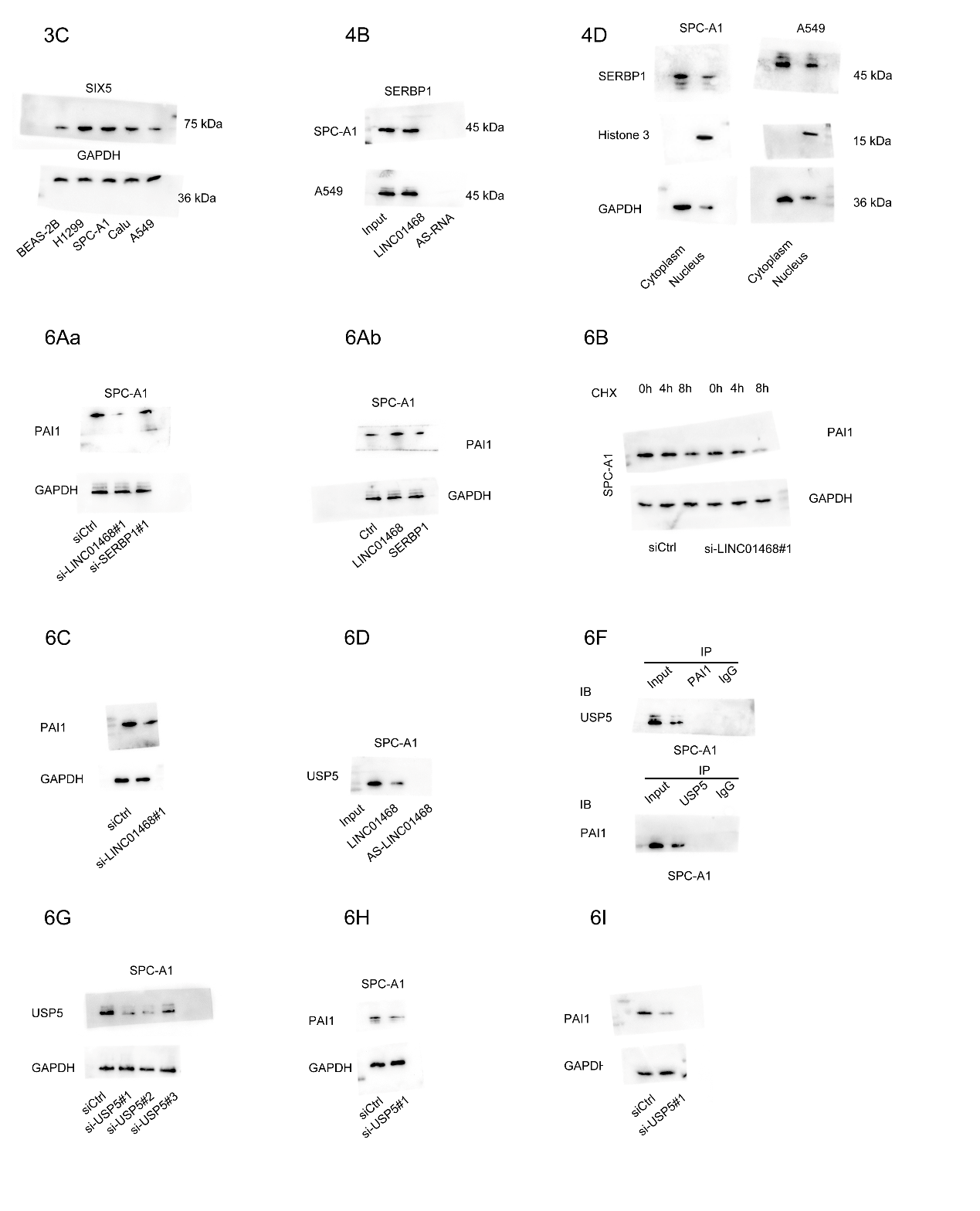

Supplement: Supplementary file 4 — Supplemental Material [file 41419_2022_4717_MOESM4_ESM.docx]

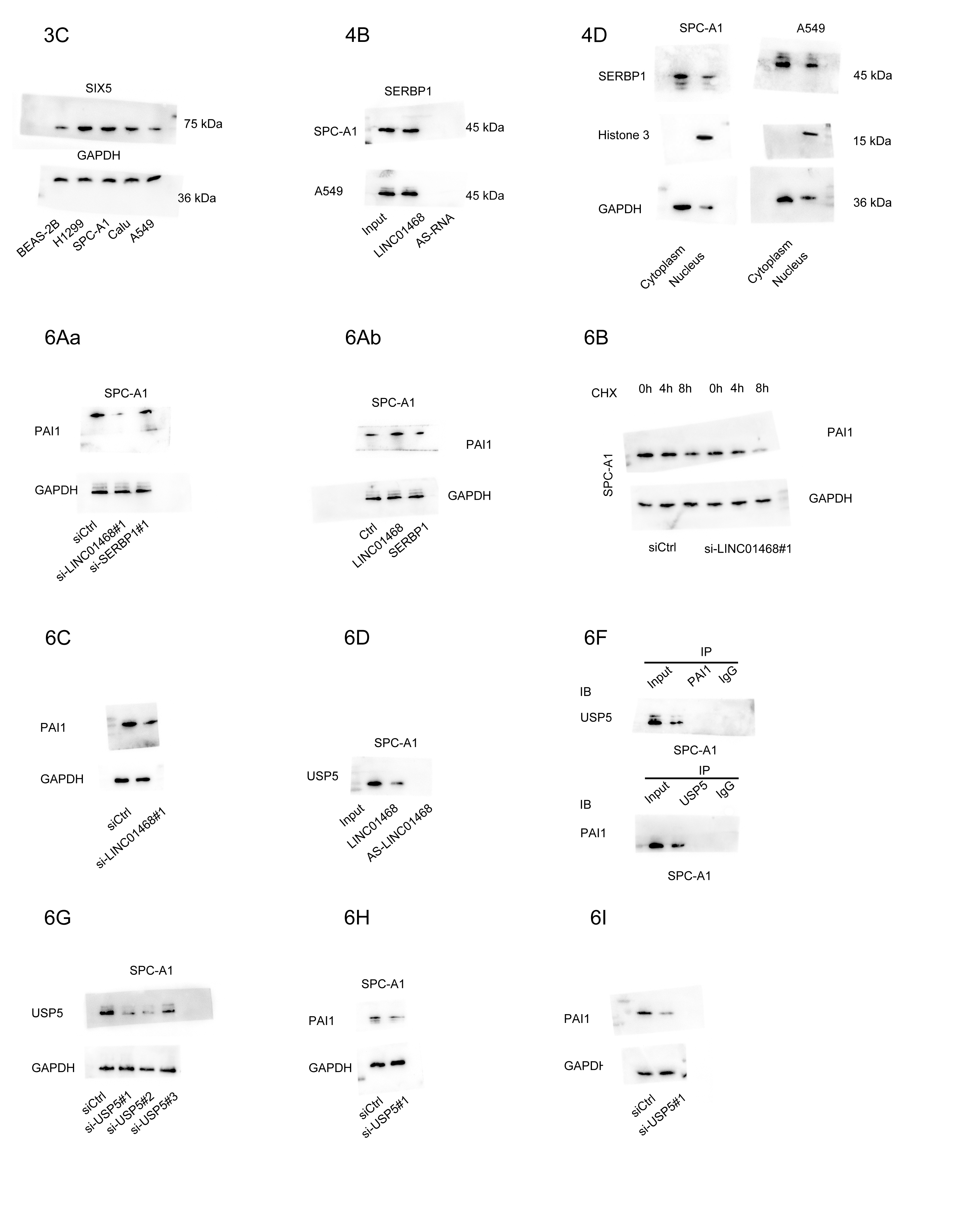

Supplement: Supplementary file 5 — Supplemental Material [file 41419_2022_4717_MOESM5_ESM.tif]
